# Loop Diuretic Therapy in Severe Aortic Stenosis: Marker or Mediator of Adverse Outcomes?

**DOI:** 10.1002/clc.70264

**Published:** 2026-01-23

**Authors:** Masaki Miyazawa, Teruhiko Imamura

**Affiliations:** ^1^ Second Department of Internal Medicine University of Toyama Toyama Japan

**Keywords:** aortic stenosis, congestion, heart failure

To Editor,

The association between loop diuretic therapy (LDT), congestion, heart failure severity, and post‐procedural outcomes in patients with severe aortic stenosis (AS) undergoing aortic valve replacement (AVR) remains incompletely understood. In this well‐conducted observational study, the authors demonstrated that pre‐procedural LDT was associated with more advanced cardiac remodeling, greater systemic and pulmonary congestion, unfavorable invasive hemodynamics, and increased long‐term mortality after AVR [1]. Several points merit further discussion.

Although the authors clearly demonstrated robust associations between LDT, congestion markers, and adverse outcomes [1], the causal relationship remains uncertain. It is unclear whether LDT merely reflects advanced disease severity or actively contributes to worse clinical outcomes. In other words, LDT may function as a marker rather than a mediator of poor prognosis. To better disentangle this relationship, it would be informative to compare outcomes among patients with comparable doses of loop diuretics but different congestion severity. For example, matching patients with similar doses of loop diuretics but differing pulmonary artery wedge pressure or radiographic congestion scores might help clarify whether congestion itself, rather than LDT, primarily drives prognosis.

A substantial proportion of patients receiving LDT still exhibited elevated filling pressures [1], suggesting suboptimal congestion control. If congestion is the principal determinant of poor outcomes [2], more aggressive or optimized decongestive strategies—rather than avoidance of LDT—might theoretically improve hemodynamics and prognosis. This perspective is particularly relevant given the paradoxical positive association between loop diuretic dose and filling pressures observed in the study [1].

Another hypothesis is that LDT is a major driver of worse clinical outcomes. Background‐matched comparisons between patients receiving LDT and those not receiving LDT would further strengthen causal inference. Because LDT prescription was left entirely to the discretion of treating clinicians [1], confounding by indication is unavoidable (i.e., patients with more severe congestion tend to receive LDT). Although the authors appropriately acknowledge this limitation, advanced statistical approaches such as propensity score matching or inverse probability weighting could provide additional insights into whether LDT independently contributes to mortality risk beyond reflecting advanced cardiac damage.

If LDT is rather the major driver of worse clinical outcomes, the potential role of alternative or adjunctive therapies deserves consideration. If LDT is associated with renal dysfunction or neuro‐hormonal activation, partial substitution with other agents—such as vasopressin V2 receptor antagonists or sodium–glucose cotransporter‐2 inhibitors—might allow effective decongestion while preserving renal function and potentially improving outcomes [3]. Prospective studies are warranted to explore whether such strategies could modify the risk profile of patients with severe AS who require diuretic therapy prior to AVR.

## Funding

The authors received no specific funding for this work.

## Ethics Statement

The authors have nothing to report.

## Consent

The authors have nothing to report.

## Conflicts of Interest

The authors declare no conflicts of interest.

## Data Availability

The manuscript does not include any original data.
